# Design of MMP-1 inhibitors via SAR transfer and experimental validation

**DOI:** 10.1038/s41598-022-25079-4

**Published:** 2022-12-03

**Authors:** Kohei Umedera, Atsushi Yoshimori, Jürgen Bajorath, Hiroyuki Nakamura

**Affiliations:** 1 School of Life Science and Technology, Tokyo Institute of Technology, 4259, Nagatsuta-Cho, Midori-Ku, Yokohama, 226-8503 Japan; 2 Institute for Theoretical Medicine, Inc., 26-1, Muraoka-Higashi 2-Chome, Fujisawa, Kanagawa 251-8555 Japan; 3 Department of Life Science Informatics, B-IT, LIMES Program Unit Chemical Biology and Medicinal Chemistry, Rheinische Friedrich-Wilhelms-Universität, Friedrich-Hirzebruch-Allee 5/6, 53115 Bonn, Germany; 4 Laboratory for Chemistry and Life Science, Institute of Innovative Research, Tokyo Institute of Technology, 4259, Nagatsuta-Cho, Midori-Ku, Yokohama, 226-8503 Japan

**Keywords:** Computational biology and bioinformatics, Drug discovery

## Abstract

New matrix metalloproteinase 1 (MMP-1) inhibitors were predicted using the structure–activity relationship (SAR) transfer method based on a series of analogues of kinesin-like protein 11 (KIF11) inhibitors. Compounds **5**–**7** predicted to be highly potent against MMP-1 were synthesized and tested for MMP-1 inhibitory activity. Among these, compound **6** having a Cl substituent at the R^1^ site was found to possess ca. 3.5 times higher inhibitory activity against MMP-1 than the previously reported compound **4**. The observed potency was consistent with the presence of an SAR transfer event between analogous MMP-1 and KIF11 inhibitors. Pharmacophore fitting revealed that the higher inhibitory activity of compound **6** compared to compound **4** against MMP-1 might be due to a halogen bond interaction between the Cl substituent of compound **6** and residue ARG214 of MMP-1.

## Introduction

Investigation of structure activity relationships (SARs) is a key process during lead optimization in medicinal chemistry. Through SAR analysis, critical substitution sites in compounds are often identified and further explored chemically to increase the potency of analogues^1,2^.

For series of analogues with corresponding substituents (R-groups) that are active against the same or different targets similar SAR trends might be observed, which is also referred to as SAR transfer^3,4^. Analogue series (ASs) representing such SAR transfer events contain different core structures and pairs of analogues having the same R-groups at corresponding substitution sites^3^. Although parallel potency progression of ASs might predominantly be anticipated for the same target, consistent with the formation of corresponding compound-target interactions following scaffold replacement, SAR transfer events are also frequently found for ASs with activity against different targets^4,5^.

To systematically search for SAR transfer events involving the same or different targets, we have recently developed a computational method for the automated identification and alignment of ASs with corresponding potency progression^5^. This approach relies on fragment similarity scoring in combination with dynamic programming. The alignment of ASs with SAR transfer potential across different targets might also enable the prediction of new analogues with increased potency against a target of interest (termed SAR transfer analogues).

In this paper, we report the SAR transfer-based prediction and experimental verification of new matrix metalloproteinase 1 (MMP-1) inhibitors^6^ based on an alignment of two series of inhibitors with activity against MMP-1 and kinesin-like protein 11 (KIF11), respectively^7^. MMP-1 is one of the important collagenase families for degrading native collagen and known to play a central role in all major stages of tumor progression^8,9^. KIF11, on the other hand, is a member of the kinesin superfamily and is known as a nanomotor that moves along microtubule in cells^10–12^. Since these two proteins have completely different structures and functions, and inhibitors of these targets are therefore principally unrelated. However, we have identified an SAR transfer event involving series of inhibitors with activity against these targets, on the basis of which a new potent MMP-1 inhibitor was designed.

## Results and discussion

**Figure 1:**
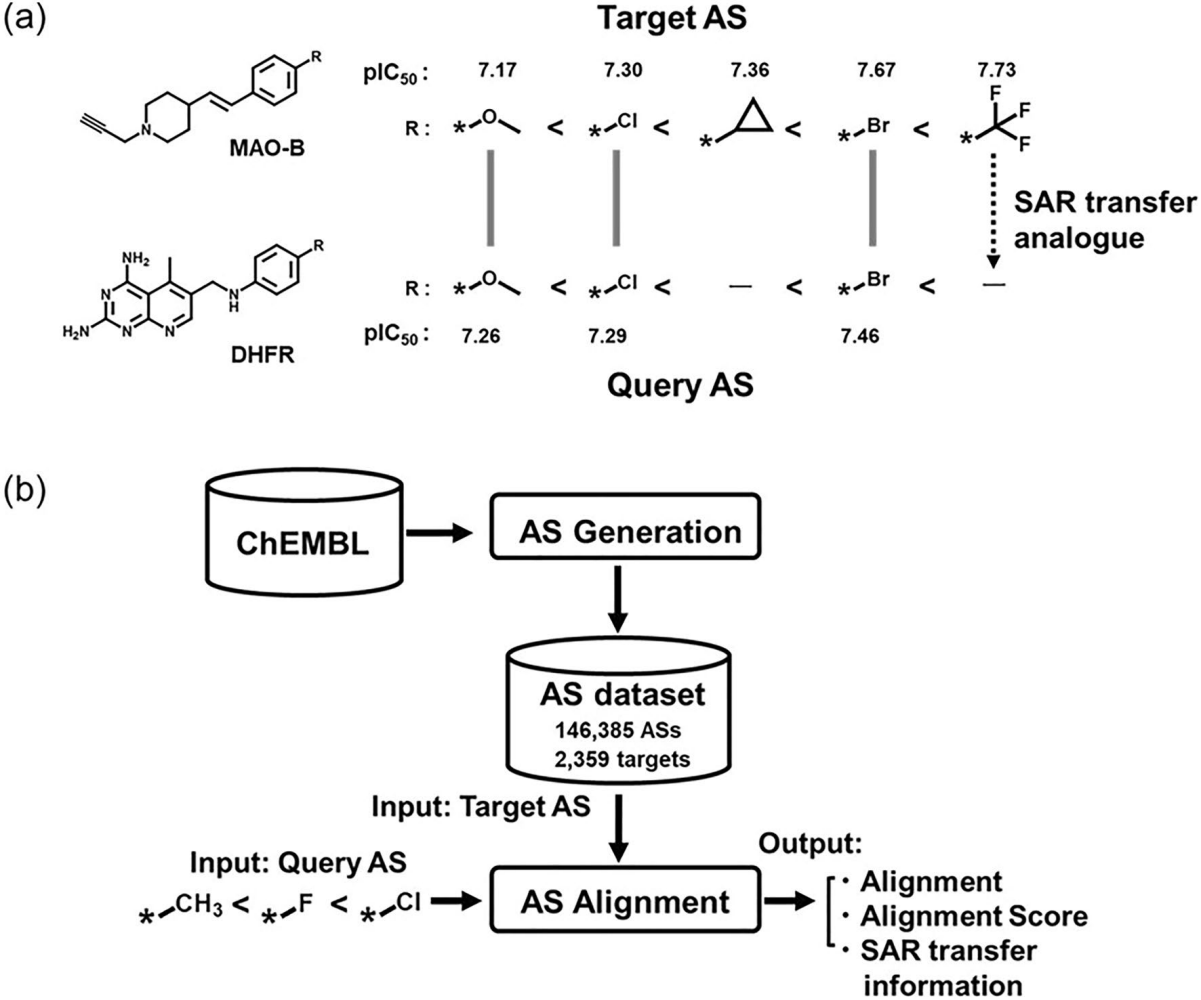
(**a**) An alignment of two exemplary ASs with corresponding pairs of analogues and activity against monoamine oxidase type B (MAO-B) and dihydrofolate reductase (DHFR) is shown. The dashed arrow indicates a possible SAR transfer analogue for the query AS. (**b**) Workflow for detection of SAR transfer analogue with AS alignment.

Figure 1a shows an exemplary SAR transfer event involving two different proteins. A query AS with activity against a target protein of interest was searched against a database of ASs with diverse activity aiming to find target ASs with SAR transfer potential. In query and target ASs, analogues with corresponding R-groups were aligned and SAR transfer was detected if there was corresponding potency progression along the aligned series. In this case, the target AS contained a trifluoromethyl analogue with highest potency that was absent in the query AS. Considering the observed SAR progression, the corresponding trifluoromethyl analogue in the query represented a possible SAR transfer analogue that might have high potency against the target of interest and expected to be highly active against the target of query AS. The systematic search for SAR transfer events and corresponding AS alignments^5^ is illustrated in Fig. 1b. The AS database contained a total of 146,385 ASs with activity 2359 targets. These ASs were algorithmically extracted from publicly available active compounds. Alignments between target and query AS were generated by dynamic programing using fragment similarity between ASs as indicator^5^.

We previously demonstrated the prediction of an MMP‑1 inhibitor activity cliff using the SAR matrix (SARM) approach^13^. An activity cliff consists of a pair of structural analogues with a large difference in potency. The SARM methodology was developed for systematic analysis of SAR data and prediction of virtual analogues of known active compounds. SARM organizes ASs and associated SAR information in matrices based on structural relationships between series, revealing activity cliffs^14–16^. Hence, the SARM approach is conceptually distinct from SAR transfer-based analogue design. The SARM-based activity cliff application predicted compound **4** as a novel combination of a core structure and substituent extracted from structurally distinct inhibitors. Indeed, compound **4** was found to exhibit 60-fold higher potency than its oxapyrrolidine skeleton-based analogue compound **3**^17,18^.

**Figure 2:**
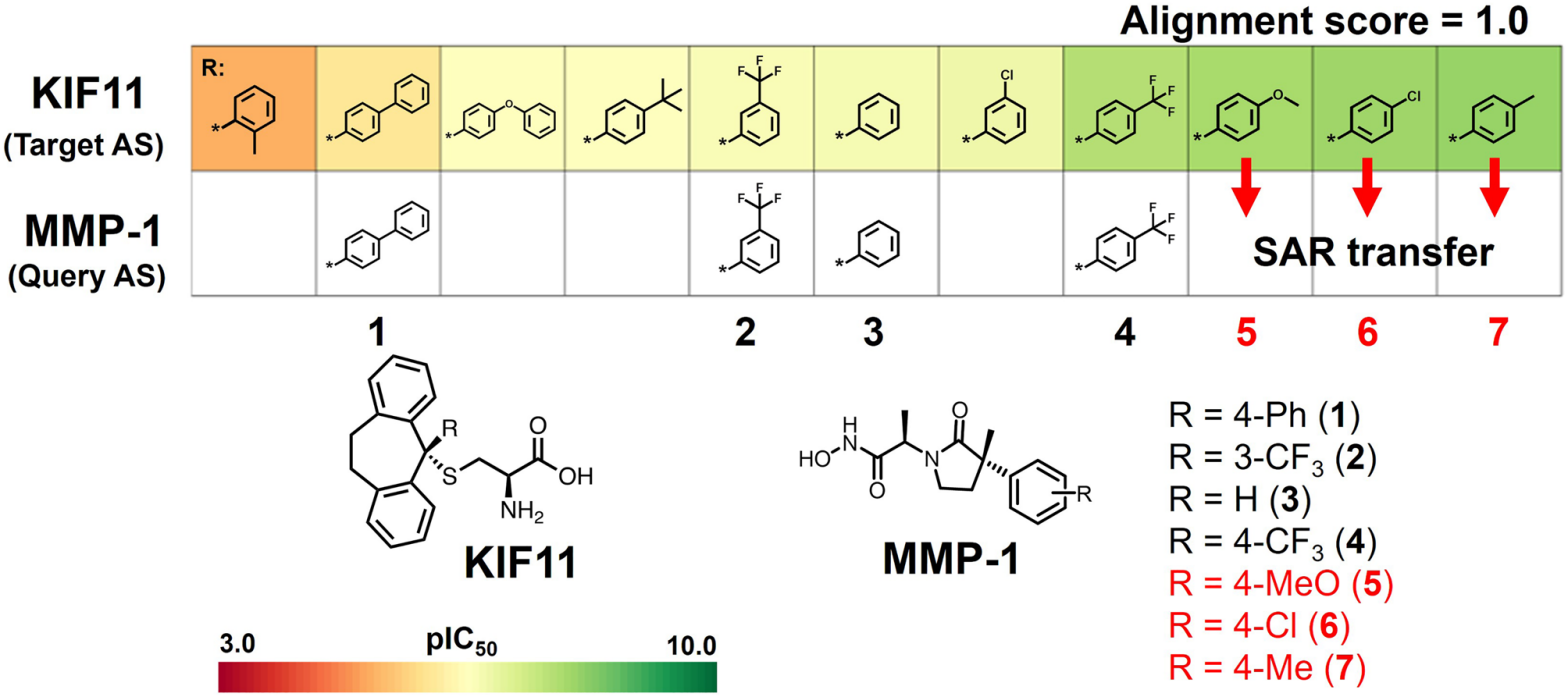
Analogue series alignment of KIF11 and MMP-1 inhibitors and SAR transfer from KIF11 to MMP-1.

In this study, we further extended MMP-1 inhibitor design by searching a query AS of MMP-1 inhibitors containing compound **4** for target ASs with SAR transfer potential in our previously reported AS database. In our systematic search, the best AS alignment score was obtained for a target AS with activity against KIF11, as shown in Fig. 2. Based on this alignment, three analogues with different R-groups were suggested as SAR transfer analogies and candidates for potent inhibition of MMP-1 (compounds **5**–**7** in Fig. 2).

**Figure 3:**
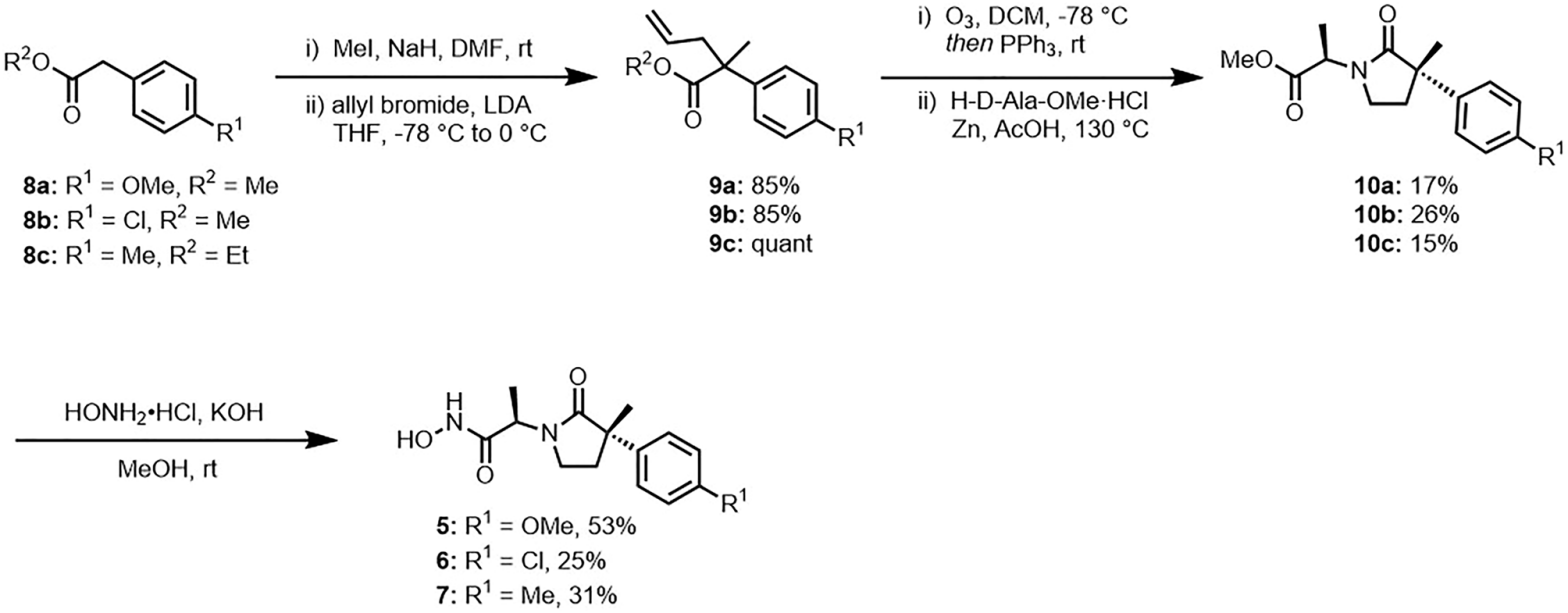
Synthesis of compounds **5**–**7.**

Figure 3 shows the synthesis of compounds **5**–**7**^18^. The commercially available esters **8a**–**c** were chosen as starting materials and treated with sodium hydride in *N,N*-dimethylformamide (DMF) to introduce methyl and allyl groups stepwise at α position of each ester. The resulting allylic esters **9a**–**c** were subjected to ozonolysis followed by reductive amination with D-alanine methyl ester in the presence of zinc dust in acetic acid under reflux conditions to afford 1:1 diastereomer mixtures of the corresponding γ-lactams **9a**–**c**. The desired stereoisomers **9a**–**c**, which were eluted after the first undesired stereoisomers separated by silica gel column chromatography, were treated with NH_2_OH under basic conditions to give *N*-hydroxyamides **5**–**7** in 25–53% yields. It is known that the (*S*)-configuration at the quaternary carbon center of a series of compounds, such as compounds **3** and **4**, is essential for the MMP-1 inhibition^18^.

Next, the inhibitory activity of the synthesized compounds **5**–**7** was examined against MMP-1 using a colorimetric assay according to the manufacturer’s protocols of the MMP-1 colorimetric drug discovery kit (ENZ, BML-AK404-0001). Briefly, the reaction was started by the addition of the MMP-1 substrate. The absorbance of each well was measured at A_412nm_ using a microplate reader. As shown in Table 1, the IC_50_ value of compound **4** (R^1^ = CF_3_) reported previously^13^ was 0.12 ± 0.01 µM, whereas compounds **5** (R^1^ = OMe) and **7** (R^1^ = Me), which represented potential SAR transfer analogues, exhibited slightly lower inhibitory activity than compound **4**: IC_50_ values for compounds **5** and **7** are 0.36 ± 0.04 and 0.50 ± 0.023 µM, respectively). However, the inhibitory activity of compound **6**, which had a Cl substituent at the R^1^ position, exhibited the highest inhibitory activity among a series of oxapyrrolidine analogues with IC_50_ of 0.034 ± 0.026 µM, indicating that the Cl substituent was more favorable than the CF_3_ group of compound **4**. The 4-Cl substituted phenyl side chain was the most potent KIF11 inhibitor. Thus, SAR transfer-based prediction facilitated the design of potent MMP-1 inhibitors on the basis of a KIF11 inhibitor target AS.

**Table 1:** MMP-1 inhibitory activity of compounds **4**–**7.**

Compound	R^1^	IC_50_ (µM)^a^
**4**^b^	CF_3_	0.12 ± 0.01
**5**	OMe	0.36 ± 0.04
**6**	Cl	0.034 ± 0.026
**7**	Me	0.50 ± 0.023

^a^The compound concentration required for 50% inhibition (IC_50_) was determined from semi-logarithmic dose–response plots, and the results represent the mean ± SD of triplicated samples.

^b^Previously reported compound^13^.

**Figure 4:**
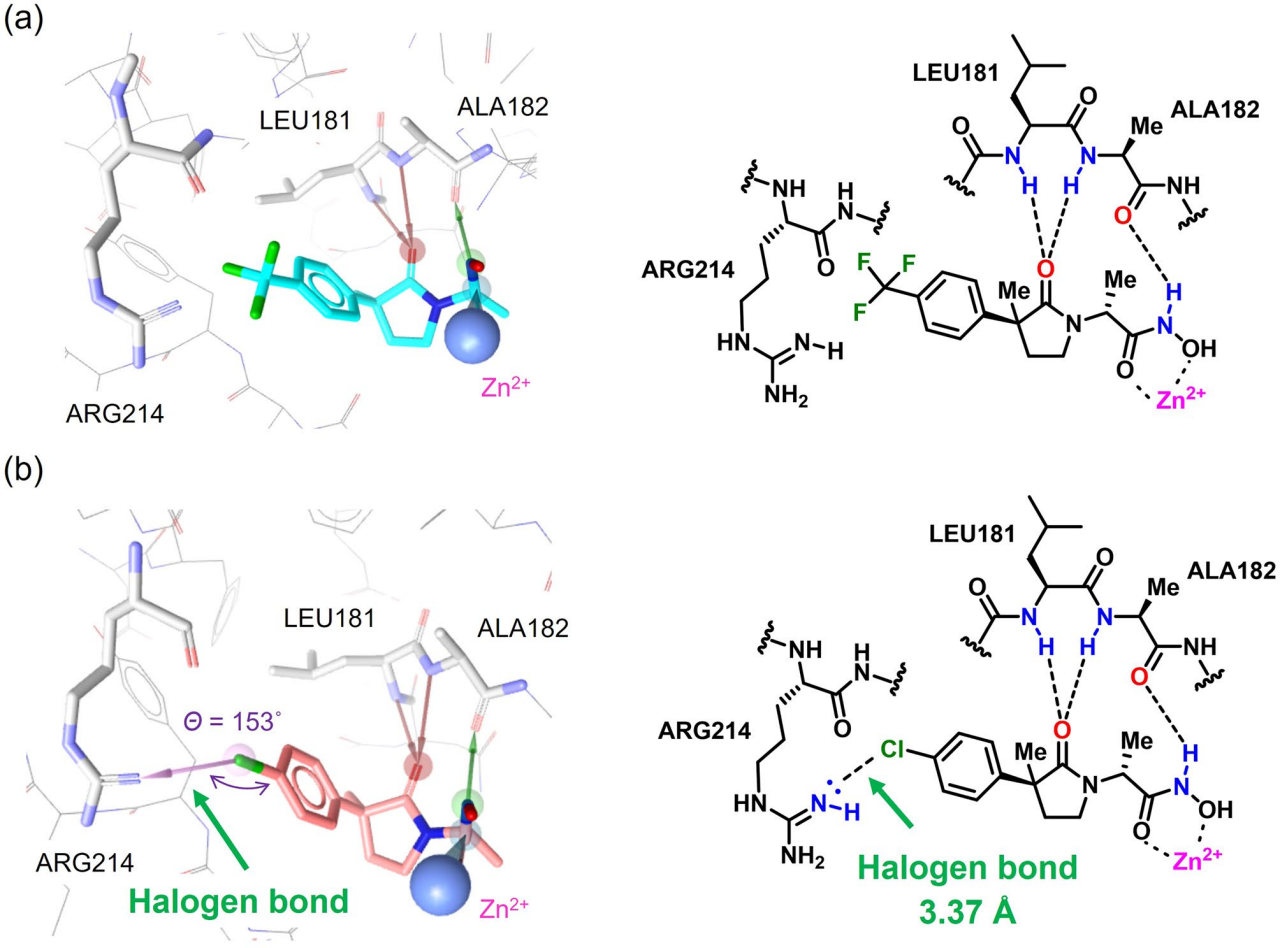
Pharmacophore fitting of compound (**a**) **4** and (**b**) **6** to the SC44463 pharmacophore model constructed from the crystal structure of compound SC44463 in complex with MMP-1 (PDB entry 1FBL)^19^.

In order to investigate possible reasons for the improved inhibitory activity of compound **6** against MMP-1, compounds **4** and **6** were examined using pharmacophore model constructed from the crystal structure of compound SC44463 in complex with MMP-1 (PDB entry 1FBL)^18,19^. In the resulting pharmacophore fit, the carbonyl group of the γ-lactam was indicated to contribute to MMP-1 inhibitory activity through hydrogen bond formation with the backbone of residues LEU181 and ALA182 (Fig. 4a)^20^. The trifluoromethyl phenyl group of compound **4** was placed into the hydrophobic S1’ pocket located by the isobutyl group of SC44463. Moreover, for compound **6**, a new halogen bond interaction was identified between the Cl substituent of compound **6** and residue ARG214 of MMP-1 with a calculated distance of 3.37 Å with an angle of 153° (N…Cl-Ar), providing a plausible reason for the increased inhibitory activity of compound **6** against MMP-1 (Fig. 4b). In medicinal chemistry, halogen bonding has been recognized as an important affinity comparable to hydrogen bonding and is expected to enhance binding affinity in drug design^21^. Therefore, it would be expected that changing Cl at the R^1^ group of compound **6** to Br or I further increases the binding affinity, resulting in creation of compounds with high inhibitory activity.

## Conclusion

Herein, we have investigated SAR transfer-based prediction of MMP-1 inhibitors. In a systematic search using a query AS of known MMP-1 inhibitors, a target AS of KIF11 inhibitors has been detected enabling the design of SAR transfer analogues for MMP-1. Among the candidate compounds generated on the basis of the AS alignment, compound **6** was found to exhibit ca. 3.5-fold higher potency than its analogue compound **4**, which was previously identified as a potent MMP-1 inhibitor by SAR matrix analysis. Pharmacophore modeling of compounds **4** and **6** revealed a halogen bond between the Cl substituent of compound **6** and residue ARG214 of MMP-1 as a likely distinguishing interaction, suggesting that it might be the reason for the increased inhibitory activity of compound **6** against MMP-1. Since the 4-Cl substituted phenyl side chain was the most potent KIF11 inhibitor, the SAR transfer analysis successfully predicted a suitable R-group for MMP-1 inhibitors from a different AS. Therefore, we believe that our SAR transfer method across different targets has considerable potential for the design of potent analogues in evolving ASs with activities against diverse targets.

## Methods

### General

Compound **4** was synthesized according to the previously reported procedures^8^ and compounds **8a** and **8b,c** were purchased from Acros Organics and TCI Co. Ltd., respectively. The intermediates **9a** and **9b** were synthesized according to the literature procedure and the compound data were in accordance with those in the literature^22^.

### Synthesis of methyl 2-methyl-2-(*p*-tolyl)pent-4-enoate (9c)

A suspension of NaH 60% dispersion in Paraffin Liquid (116 mg, 2.90 mmol) in DMF (9.0 mL) was cooled to 0 °C and ethyl *p*-tolylacetate (529 µL, 3.00 mmol) was added dropwise. After being stirred for 2 h at the same temperature under an argon atmosphere, methyl iodide (187 µL, 3.00 mmol) was added, and the reaction mixture was further stirred for 1 h at room temperature. The reaction mixture was then diluted with saturated aqueous NH_4_Cl, and the aqueous layer was extracted with Et_2_O for three times. The combined organic layers were washed with brine, dried over MgSO_4,_ filtered, and concentrated under reduced pressure. The residue was passed through a pad of silica gel (hexane:EtOAc = 8:2) to afford crude ester (587 mg). To a solution of diisopropylamine (600 µL, 4.27 mmol) in THF (12 mL) was added 1.59 M *n*-BuLi hexane solution (2.45 mL, 3.90 mmol) dropwise at − 78 °C. The reaction mixture was stirred for 30 min at 0 °C under an argon atmosphere. The crude ester (587 mg) dissolved in THF (3.0 mL) was added at − 78 °C. After being stirred for 1 h at − 78 °C, the reaction mixture was added ally bromide (400 µL, 4.74 mmol) and stirred for 40 min at 0 °C. The reaction mixture was diluted with saturated aqueous NH_4_Cl, and the aqueous layer was extracted with Et_2_O for three times. The combined organic layers were washed with brine, dried over MgSO_4,_ filtered, and concentrated under reduced pressure. The residue was purified by silica gel column chromatography (hexane 100% to hexane:EtOAc = 95:5) to afford compound **9c** (688 mg, 2.96 mmol, quant. in 2 steps) as a yellow oil. ^1^H NMR (400 MHz, CDCl_3_) δ 7.22 (d, *J* = 8.3 Hz, 2H), 7.14 (d, *J* = 8.2 Hz, 2H), 5.69–5.59 (m, 1H), 5.10–5.04 (m, 2H), 4.14 (q, *J* = 7.1 Hz, 2H), 2.83 (dd, *J* = 7.4, 13.7 Hz, 1H), 2.65 (dd, *J* = 7.1, 13.7 Hz, 1H), 2.34 (s, 3H), 1.52 (s, 3H), 1.20 (t, *J* = 7.1 Hz, 1H); ^13^C NMR (100 MHz, CDCl_3_) δ 175.9, 140.7, 136.4, 134.4, 129.2, 126, 118.3, 77.5, 77.2, 76.8, 60.9, 49.6, 43.9, 22.8, 21, 14.2; IR (neat, cm^−1^): 3076, 2979, 2937, 2870, 1727, 1514, 1456, 1375, 1229, 1143, 1096, 1020, 815, 916; HRMS (ESI) calcd for C_15_H_21_O_2_^+^ [M + H^+^] 233.1536, found 233.1531.

### Synthesis of methyl (*S*)-2-(3-(4-methoxyphenyl)-3-methyl-2-oxopyrrolidin-1-yl)acetate (10a)

Ozone was pumped into a solution of ester **9a** (579 mg, 2.47 mmol) in CH_2_Cl_2_ (7.0 mL) at − 78 °C. After starting material disappeared by TLC analysis, the reaction mixture was purged with argon followed by the addition of triphenylphosphine (579 mg, 2.96 mmol). After stirred for 1 h at room temperature, the mixture was concentrated under vacuum. The residue was purified silica gel column chromatography (hexane:EtOAc = 90:10 to 80:20) to afford the crude aldehyde (394 mg). To a solution of the crude aldehyde and D-alanine methyl ester hydrochloride (257 mg, 1.84 mmol) in acetic acid (8.0 mL) was added zinc powder (1.09 g, 16.7 mmol) portion-wise. The mixture was stirred for 2.5 h at 130 °C, and then cooled to room temperature. Following addition of CH_2_Cl_2_, the mixture was passed through a pad of Celite® and the filter cake was washed with MeOH/CH_2_Cl_2_. The filtrate was concentrated under reduced pressure. The residue was purified by silica gel column chromatography (hexane:EtOAc = 80:20 to 70:30) to afford slow eluting isomer **10a** (124 mg, 0.426 mmol, 17% in 2 steps) as a yellow oil. [α]_D_^28.5^ + 10.2 (c 1.00 in CHCl_3_); ^1^H NMR (400 MHz, CDCl_3_) δ 7.33 (d, *J* = 8.8 Hz, 2H), 6.85 (d, *J* = 8.8 Hz, 2H), 4.96 (q, *J* = 7.4 Hz, 1H), 3.77 (s, 3H), 3.66 (s, 3H), 3.36–3.33 (m, 2H), 2.44–2.38 (m, 1H), 2.14–2.07 (m, 1H), 1.52 (s, 3H), 1.45 (d, *J* = 7.1 Hz, 3H); ^13^C NMR (100 MHz, CDCl_3_) δ 178.1, 171.9, 158.3, 135.7, 127.5, 113.8, 55.3, 52.3, 49.6, 48.2, 40.5, 36, 24.9, 14.8; IR (neat, cm^−1^): 3068, 3032, 2954, 2886, 2837, 1742, 1688, 1610, 1513, 1455, 1423, 1278, 1249, 1185, 1031, 832; HRMS (ESI) calcd for C_16_H_22_NO_4_^+^ [M + H^+^] 292.1541, found 292.1546.

### Synthesis of methyl (*S*)-2-(3-methyl-2-oxo-3-(4-cholorophenyl)pyrrolidin-1-yl)acetate (10b)

This compound was prepared from ester **9b** (541 mg, 2.27 mmol) using the procedure described above for **10a** to afford the slow eluting desired isomer **10b** (97.6 mg, 0.354 mmol, 2 steps 15%) as a yellow oil. [α]_D_^27.1^ − 2.9 (c 1.0 in CHCl_3_); ^1^H NMR (400 MHz, CDCl_3_) δ 7.35 (d, *J* = 8.7 Hz, 2H), 7.28 (d, *J* = 8.7 Hz, 2H), 4.94 (q, *J* = 7.4 Hz, 1H), 3.67 (s, 3H), 3.37–3.34 (m, 2H), 2.42–2.36 (m, 1H), 2.17–2.10 (m, 1H), 1.52 (s, 3H), 1.45 (d, *J* = 7.4 Hz, 3H); ^13^C NMR (100 MHz, CDCl_3_) δ 177.5, 171.8, 142.2, 132.6, 128.6, 127.9, 52.3, 49.7, 48.5, 40.5, 35.8, 24.8, 14.8; IR (neat, cm^−1^): 3026, 2969, 2952, 2928, 2876, 1718, 1690, 1653, 1514, 1455, 1364, 1277, 1214, 1179, 924; HRMS (ESI) calcd for C_15_H_18_ClNO_3_Na^+^ [M + Na^+^] 318.0867, found 318.0876.

### Synthesis of ethyl (*S*)-2-(3-methyl-2-oxo-3-(*p*-tolyl)pyrrolidin-1-yl)acetate (10c)

This compound was prepared from ester **9c** (539 mg, 2.32 mmol) using the procedure described above for **10a** to afford the slow eluting desired isomer **10c** (97.6 mg, 0.354 mmol, 2 steps 15%) as a yellow oil. [α]_D_^27.8^ + 3.0 (c 0.99 in CHCl_3_); ^1^H NMR (400 MHz, CDCl_3_) δ 7.30 (d, *J* = 8.2 Hz, 2H), 7.13 (d, *J* = 8.1 Hz, 2H), 4.97 (q, *J* = 7.4 Hz, 1H), 3.67 (s, 3H), 3.37–3.33 (m, 2H), 2.46–2.40 (m, 1H), 2.31 (s, 3H), 2.16–2.10 (m, 1H), 1.54 (s, 3H), 1.45 (d, *J* = 7.5 Hz, 3H); ^13^C NMR (100 MHz, CDCl_3_) δ 178, 171.9, 140.7, 136.3, 129.2, 126.3, 52.3, 49.6, 48.6, 40.5, 36.1, 24.9, 21.0, 14.9; IR (neat, cm^-1^): 3026, 2969, 2952, 2928, 2876, 1743, 1690, 1514, 1455, 1421, 1375, 1277, 1204, 1076, 818; HRMS (ESI) calcd for C_16_H_22_NO_3_^+^ [M + H^+^] 276.1594, found 276.1598.

### (*R*)-*N*-hydroxy-2-((*S*)-3-(4-methoxyphenyl)-3-methyl-2-oxopyrrolidin-1-yl)propenamide (5)

Hydroxylamine hydrochloride (738 mg, 11.4 mmol) in hot methanol (3.8 mL) was added KOH (890 mg, 15.9 mmol) dissolved in MeOH (2.2 mL). The mixture was cooled to room temperature and passed through a pad of Celite® to afford 1.9 M hydroxylamine solution. To a solution of **10a** (64.9 mg, 0.219 mmol) in MeOH (500 µL) was added hydroxylamine solution (620 µL) and stirred for 1 h at room temperature under an argon atmosphere. The reaction mixture was then diluted with water and the pH was adjusted to 5–6 with 1 N HCl at 0 °C. The aqueous layer was extracted three times with EtOAc. The combined organic layers were washed with brine, dried over MgSO_4,_ filtered, and concentrated under reduced pressure. The residue was purified by preparative TLC (hexane:EtOAc = 5:95) to afford hydroxyamide **5** (31.0 mg, 0.106 mmol, 53%) as a yellow oil. [α]_D_^28.4^ + 44.5 (c 095 in CHCl_3_); ^1^H NMR (400 MHz, CDCl_3_) δ 7.21 (d, *J* = 8.8 Hz, 2H), 6.82 (d, *J* = 8.8 Hz, 2H), 4.69 (q, *J* = 7.1 Hz, 1H), 3.74 (s, 3H), 3.42 (t, *J* = 6.5 Hz, 2H), 2.38–2.31 (m, 1H), 2.10−2.04 (m, 1H), 1.49 (s, 3H), 1.37 (d, *J* = 7.0 Hz, 3H); ^13^C NMR (100 MHz, CDCl_3_) δ 179, 167.8, 158.5, 135.2, 127.2, 114.1, 55.4, 48.5, 48.4, 41.2, 35.7, 24.6, 14.2; IR (neat, cm^−1^): 3208, 3029, 2969, 2936, 1716, 1513, 1455, 1431, 1249, 1186, 1031, 833; HRMS (ESI) calcd for C_15_H_19_N_2_O_4_^−^ [M–H^+^] 291.1350, found 291.1342.

### (*R*)-*N*-hydroxy-2-((*S*)-3-(4-chlorophenyl)-3-methyl-2-oxopyrrolidin-1-yl)propenamide (6)

This compound was prepared from ester **10b** (64.9 mg, 0.219 mmol) using the procedure described above for **5** to afford **6** (16.5 mg, 0.0556 mmol, 25%) as a yellow oil. [α]_D_^27.9^ + 56.9 (c 0.79 in CHCl_3_); ^1^H NMR (400 MHz, CDCl_3_) δ 7.24 (s, 4H), 4.69 (q, *J* = 7.04 Hz, 1H), 3.48–3.38 (m, 2H), 2.37–2.30 (m, 1H), 2.15–2.08 (m, 1H), 1.50 (s, 3H), 1.39 (d, *J* = 7.03, 3H); ^13^C NMR (100 MHz, CDCl_3_) δ 178.4, 167.8, 141.7, 132.9, 128.9, 127.6, 48.8, 48.4, 41.2, 35.5, 24.6, 14.2; IR (neat, cm^−1^): 3211, 3027, 2971, 2932, 2882, 1737, 1660, 1492, 1455, 1429, 1372, 1281, 1098, 1010; HRMS (ESI) calcd for C_14_H_16_ClN_2_O_3_^−^ [M–H^+^] 295.0855, found 295.0847.

### (*R*)-*N*-hydroxy-2-((*S*)-3-(*p*-tolyl)-3-methyl-2-oxopyrrolidin-1-yl)propenamide (7)

This compound was prepared from ester **10c** (58.3 mg, 0.20 mmol) using the procedure described above for **5** to afford **7** (18.5 mg, 0.0669 mmol, 31%) as a yellow oil. [α]_D_^28.2^ + 44.7 (c 0.84 in CHCl_3_); ^1^H NMR (400 MHz, CDCl_3_) δ 7.18 (d, *J* = 8.1 Hz, 2H), 7.10 (d, *J* = 8.0 Hz, 2H), 4.69 (q, *J* = 7.1 Hz, 1H), 3.44–3.39 (m, 2H), 2.39–2.34 (m, 1H), 2.29 (s, 3H), 2.12–2.07 (m, 1H), 1.51 (s, 3H), 1.38 (d, *J* = 7.1 Hz, 3H).; ^13^C NMR (100 MHz, CDCl_3_) δ 178.9, 167.8, 140.1, 136.6, 129.5, 126, 48.8, 48.4, 41.3, 35.7, 24.6, 21.0, 14.1; IR (neat, cm^−1^): 3208, 3024, 2969, 2926, 2873, 1737, 1660, 1514, 1455, 1430, 1373, 1279, 1216, 1022, 817; HRMS (ESI) calcd for C_15_H_19_N_2_O_3_^−^ [M–H^+^] 275.1401, found 275.1393.

### Biology

MMP-1 inhibitory assay was performed using the Matrix Metalloproteinase-1 (MMP-1) colorimetric drug discovery kit (ENZ, BML-AK404-0001) according to the manufacturer’s instructions. A known MMP-1 inhibitor, 2-[(2-Methylpropyl)[(4-methoxyphenyl)sulfonyl]amino]acetohydroximic acid (NNGH), was used as a positive control of the assay. Test inhibitors (10 mM in DMSO) were diluted at desired concentration in assay buffer. NNGH was diluted at 1/200 in assay buffer. MMP substrate was diluted at 1/25 in assay buffer. MMP-1 enzyme was diluted at 1/40 in assay buffer. After appropriate amount of assay buffer was pipetted into each desired well, prepared solutions of test inhibitors (20 µL, final concentrations: 0.014–10 µM), NNGH (20 µL), and MMP-1 (20 µL) were added to appropriate wells. The microplate was incubated for 30 min at 37 °C. The 10 µL of the prepared MMP-1 substrate solution was added into each well to allow the reaction start. The absorbance of the wells was measured at A_412nm_ using a microplate reader every minute for data analysis.

### Pharmacophore fitting

A pharmacophore model was constructed from the crystal structure of the SC44463/MMP-1 complex (PDB: 1FBL) using LigandScout 4.4 (InteLigand GmbH) for prediction of the binding interaction between compounds and MMP-1. Four pharmacophore features of SC44463 were used including a hydrogen bond acceptor (HA), hydrophobic (Hy) site, negative ionizable site, and zinc binding site/location feature (ZL). The scoring function was set to ‘Relative Pharmacophore-Fit’. After test compounds were fit to the SC44463 pharmacophore model, their interaction energy with MMP-1 was minimized.

### Detection of SAR transfer analogue using AS alignment

The details of the SAR transfer methodology have been published elsewhere^5^. Briefly, the procedure consists of two steps of AS database construction and AS alignment (Fig. 1b). Compounds with activity data were obtained from ChEMBL (version 29)^23^. Compounds from each assay of a target protein were fragmented by cleaving exocyclic single bonds applying the Hussain and Rea algorithm^24^ implemented in RDKit^25^. The value fragments (substituents) consisted of up to 14 non-hydrogen atoms, and up to 40% of the non-hydrogen atoms of the source compound were allowed. Key (scaffold) and value (substituent) fragments were stored in an index table. All source compounds associated with the same key fragment (scaffold) form an AS with a single substitution site. ASs were generated from each assay of a target protein if they contained at least three analogues. On the basis of these selection criteria, AS dataset consisting of 146,385 ASs originated from 2359 target proteins. AS alignment enabled systematic search calculations in the AS dataset. In order to align between target AS and query AS, AS alignment is achieved by Needleman-Wunsch dynamic programing^26^. Alignment scores range from 0 to 1. In the alignment, the dashed arrow on the right indicates the generation of an SAR transfer analogue for the query AS (Fig. 1a).

## Supplementary Information

Supplementary Information 1.

Supplementary Information 2.

## Data Availability

The AS dataset used in this study, ^1^H and ^13^C NMR spectral data and purity analysis for synthesized compounds, and concentration-dependent MMP1 inhibitory activity are available in Supplementary Information. The AS dataset was generated from ChEMBL (https://www.ebi.ac.uk/chembl/).
