# Supplementary material for: Design of MMP-1 inhibitors via SAR transfer and experimental validation
Source: Sci Rep. 2022 Dec 3;12:20915. doi: 10.1038/s41598-022-25079-4 (PMC9719525; doi:10.1038/s41598-022-25079-4)
Supplement: Supplementary file 1 — Supplementary Information 1. [file 41598_2022_25079_MOESM1_ESM.pdf]

Supplementary Information

Design of MMP-1 Inhibitors via SAR Transfer and Experimental Validation

Kohei Umedera,[a] Atsushi Yoshimori,[b] Jürgen Bajorath,*[c] Hiroyuki Nakamura*[a,d]

[a] School of Life Science and Technology, Tokyo Institute of Technology, Nagatsuta-cho, Midori-ku, Yokohama 226-8503, Japan

[b] Institute for Theoretical Medicine, Inc., Fujisawa, Kanagawa 251-8555, Japan

[c] Department of Life Science Informatics, B-IT, LIMES Program Unit Chemical Biology and Medicinal Chemistry, Rheinische Friedrich-Wilhelms-Universität, Endenicher Allee 19c, Bonn D-53115, Germany

[d] Laboratory for Chemistry and Life Science, Institute of Innovative Research, Tokyo Institute of Technology, Nagatsuta-cho, Midori-ku, Yokohama 226-8503, Japan

List of contents

1. The AS dataset used for SARM transfer S2

2. ^{1}H and ^{13}C NMR spectra of compounds

Compound **9c** S3

Compound **10a** S4

Compound **10b** S5

Compound **10c** S6

Compound **5** S7

Compound **6** S8

Compound **7** S9

3. Purity analysis of tested compounds **5**-**7** by HPLC S10-S11

4. MMP1 inhibitory activity of compounds **5**-**7** at tested concentrations S11

1. The AS dataset used in SARM transfer

Data structure:

ASSAYID_KEYSMILES[Tab]VALSMILES_pIC50[Tab].....

ASSAYID: identifier

KEYSMILES: core structure

VALSMILES: substituent

Attachment point: [At]

AS: analogue series

If substituents were associated with the same pIC50 value, the AS was ordered as follows:

Input AS:

[(9.2, 'Br[At]'), (5.2, 'F[At]'), (6.3, 'Cl[At]'), (6.3, 'C[At]')]

Ordered AS:

[(5.2, 'F[At]'), (6.3, 'C[At]'), (6.3, 'Cl[At]'), (9.2, 'Br[At]')]

If the first value (pIC50) in the tuples was identical, the tuples were ordered using a python script based on the second value (SMILES string).

2. NMR spectra of Compounds

Ethyl 2-methyl-2-(*p*-tolyl)pent-4-enoate (9c)

^{1}H (400 MHz, CDCl$_3$)

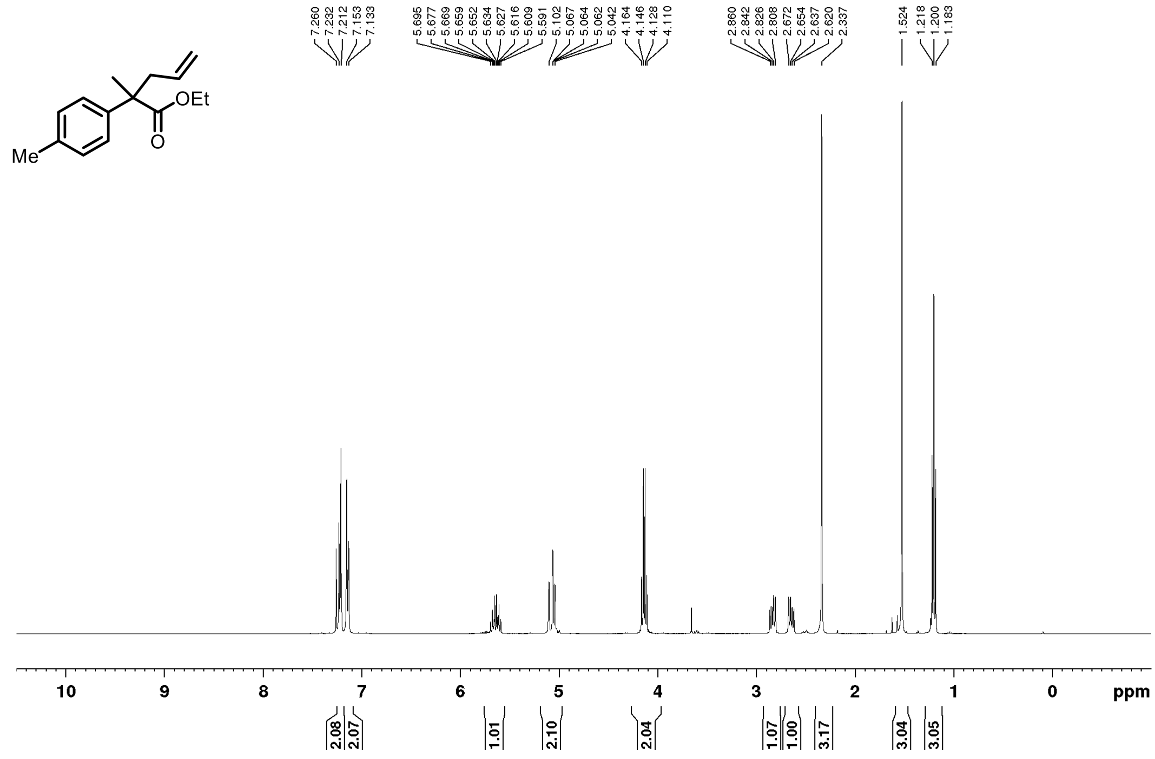

^{13}C (100 MHz, CDCl$_3$)

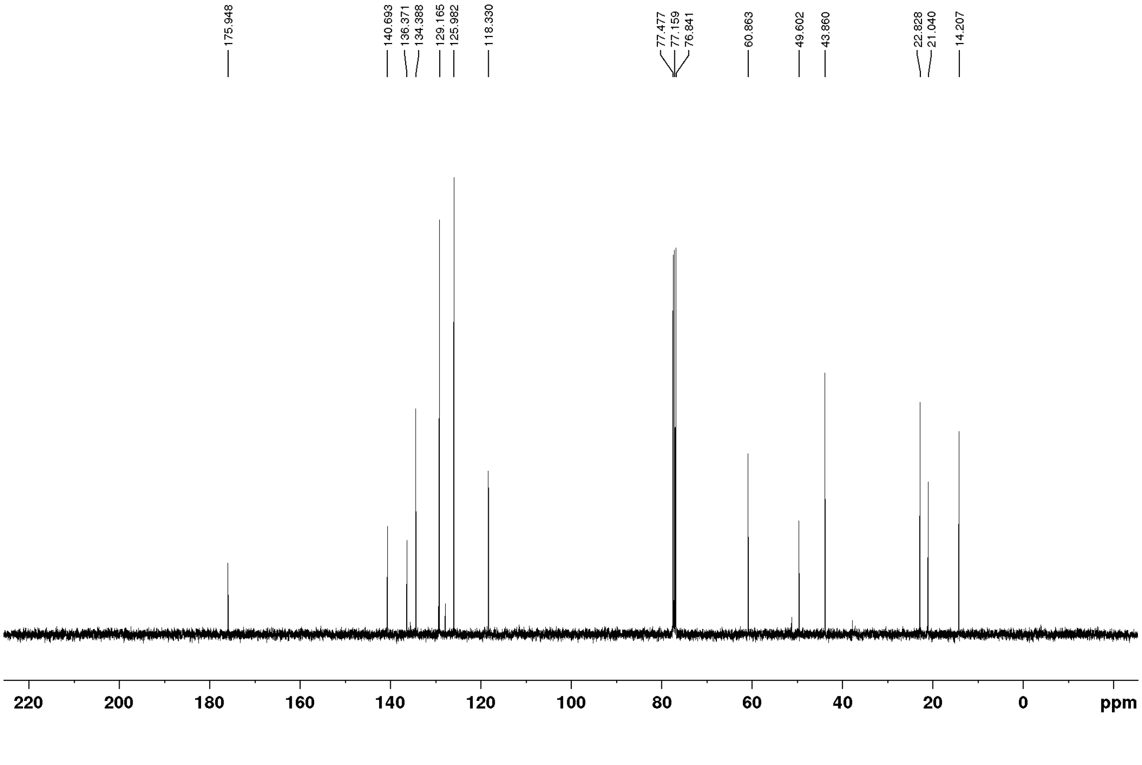

Methyl (*S*)-2-(3-(4-methoxyphenyl)-3-methyl-2-oxopyrrolidin-1-yl)acetate (10a)

^{1}H (400 MHz, $CDCl_3$)

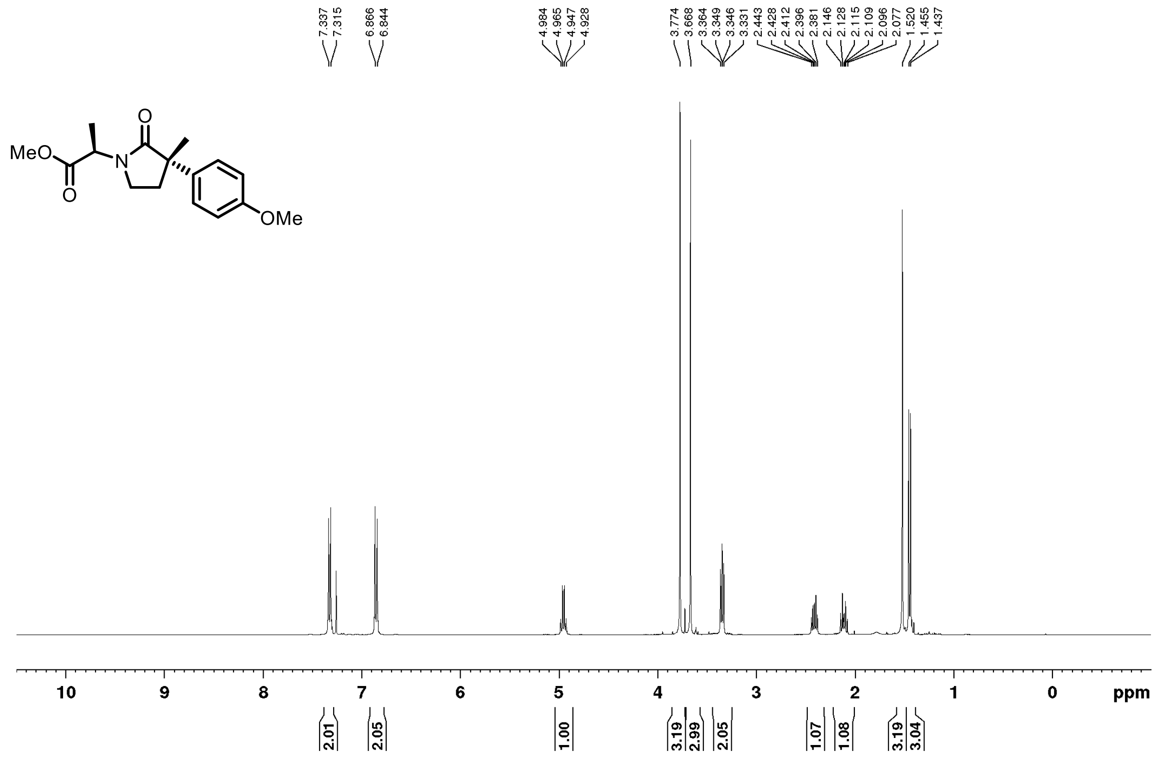

^{13}C (100 MHz, $CDCl_3$)

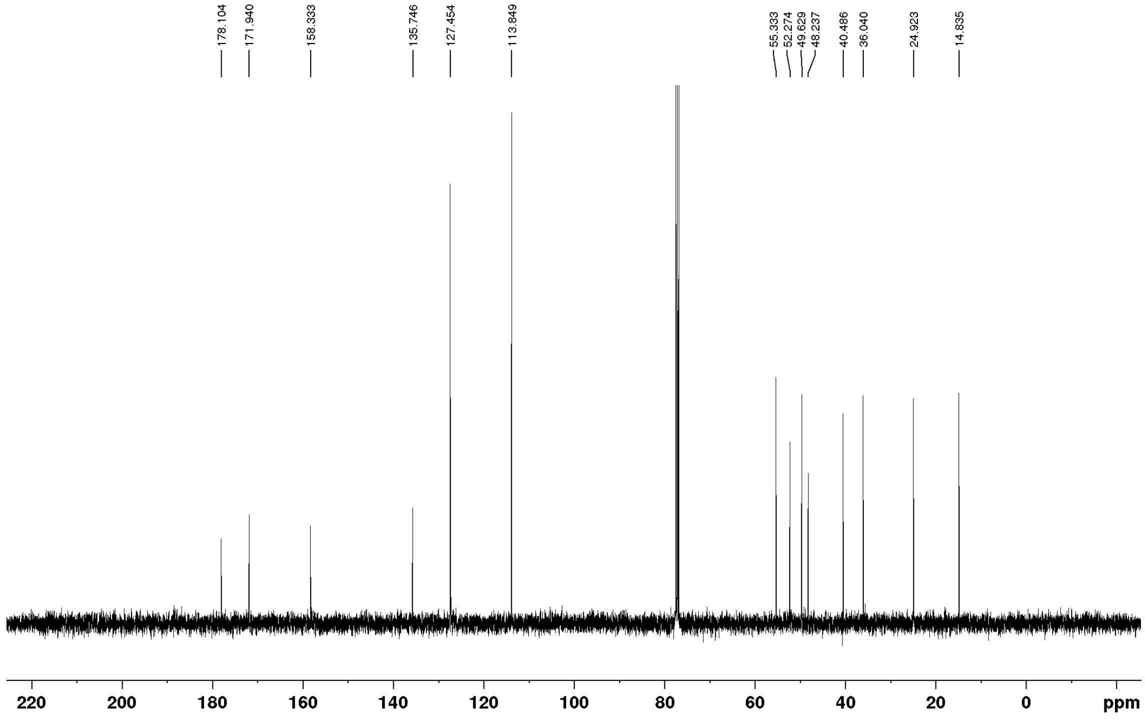

Methyl (*S*)-2-(3-methyl-2-oxo-3-(4-cholorophenyl)pyrrolidin-1-yl)acetate (10b)

^{1}H (400 MHz, $CDCl_3$)

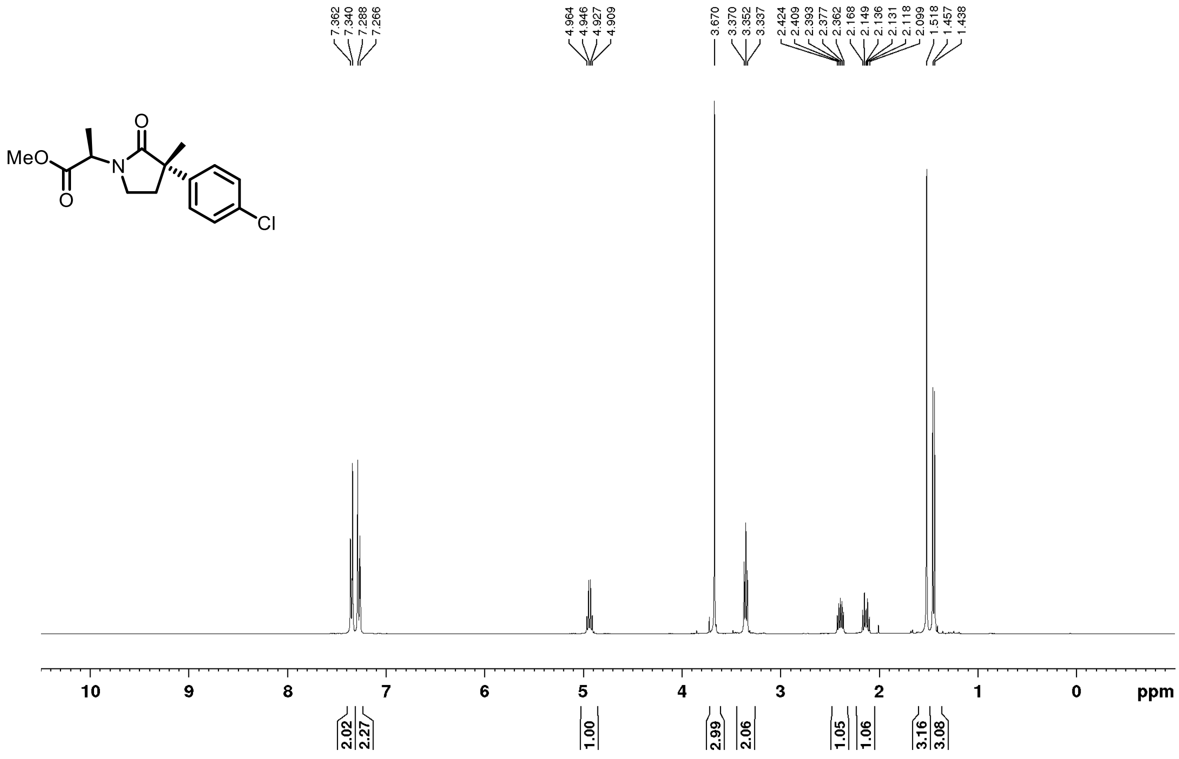

^{13}C (100 MHz, $CDCl_3$)

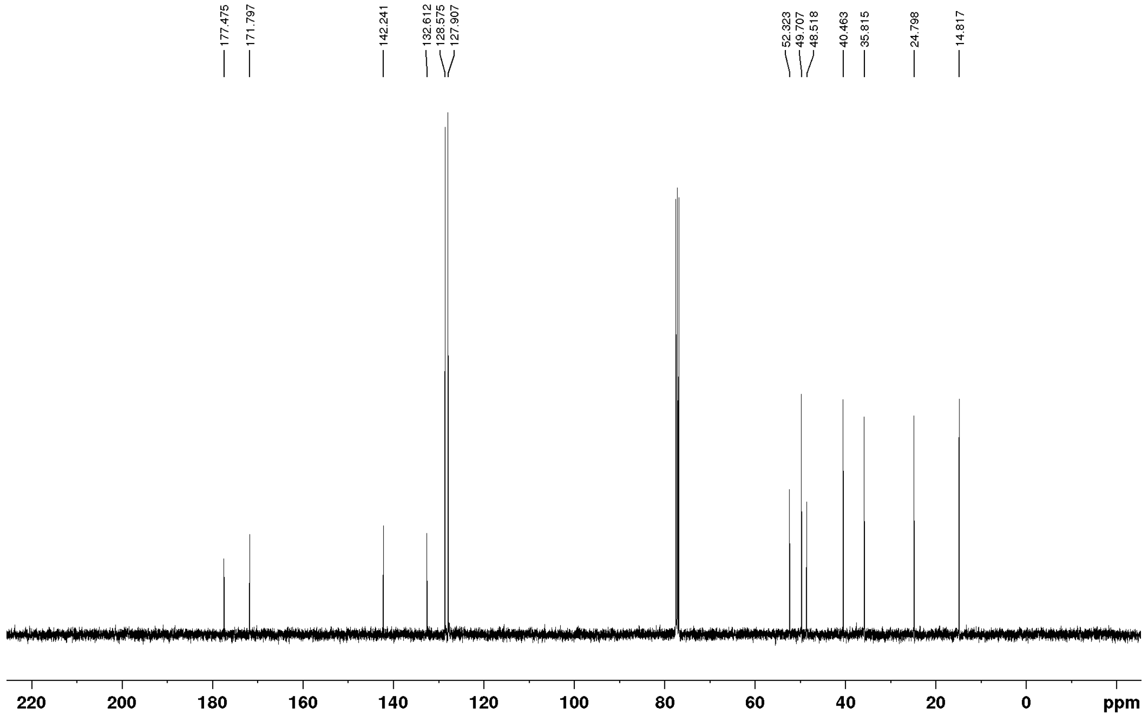

Methyl (*S*)-2-(3-methyl-2-oxo-3-(*p*-tolyl)pyrrolidin-1-yl)acetate (10c)

^{1}H (400 MHz, $CDCl_3$)

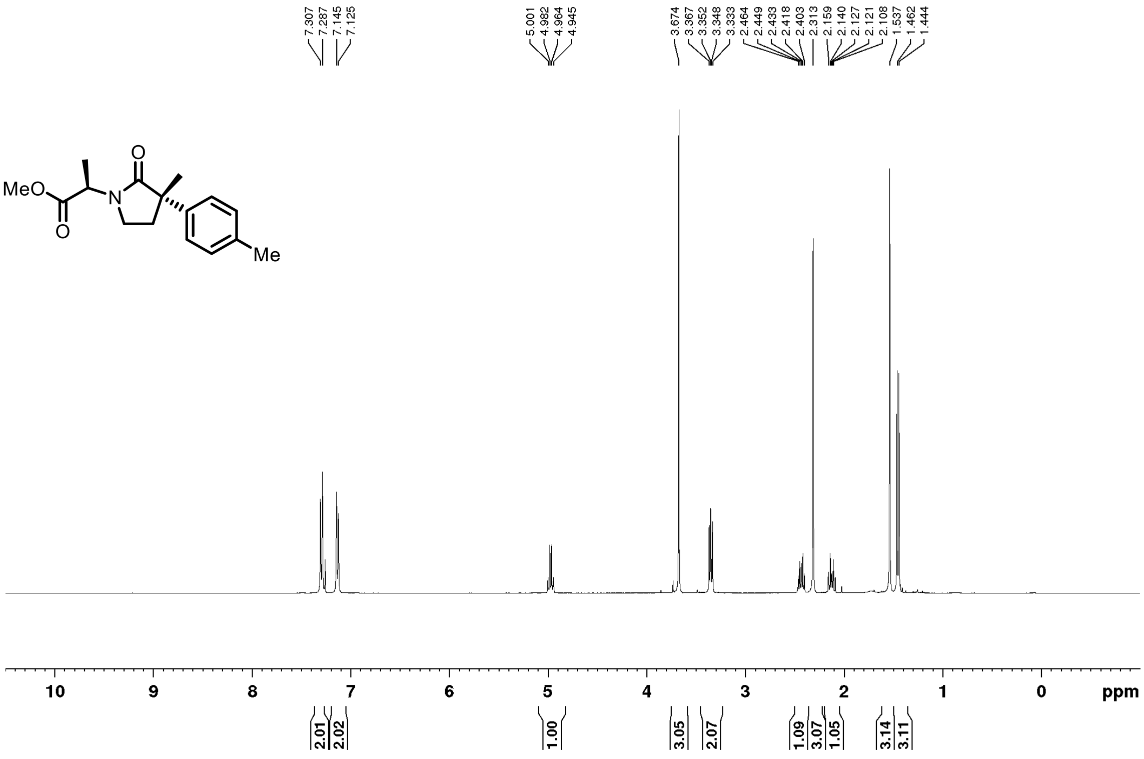

^{13}C (100 MHz, $CDCl_3$)

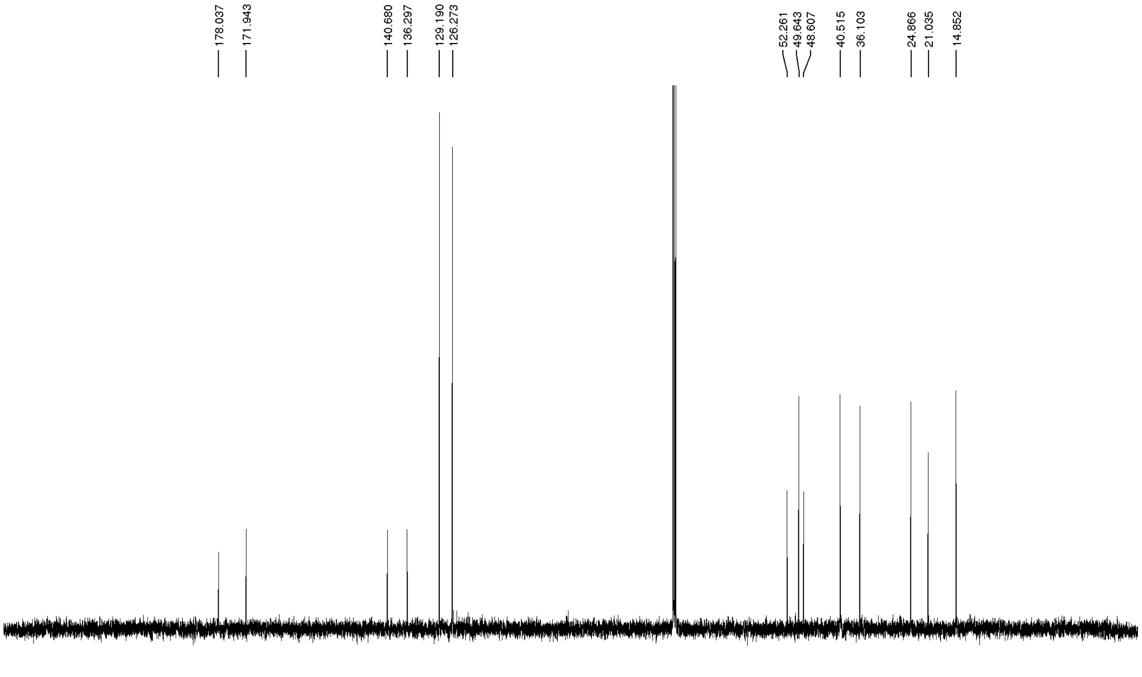

(*R*)-*N*-hydroxy-2-((*S*)-3-(4-methoxyphenyl)-3-methyl-2-oxopyrrolidin-1-yl)propenamide (5)

^{1}H (400 MHz, CDCl$_3$)

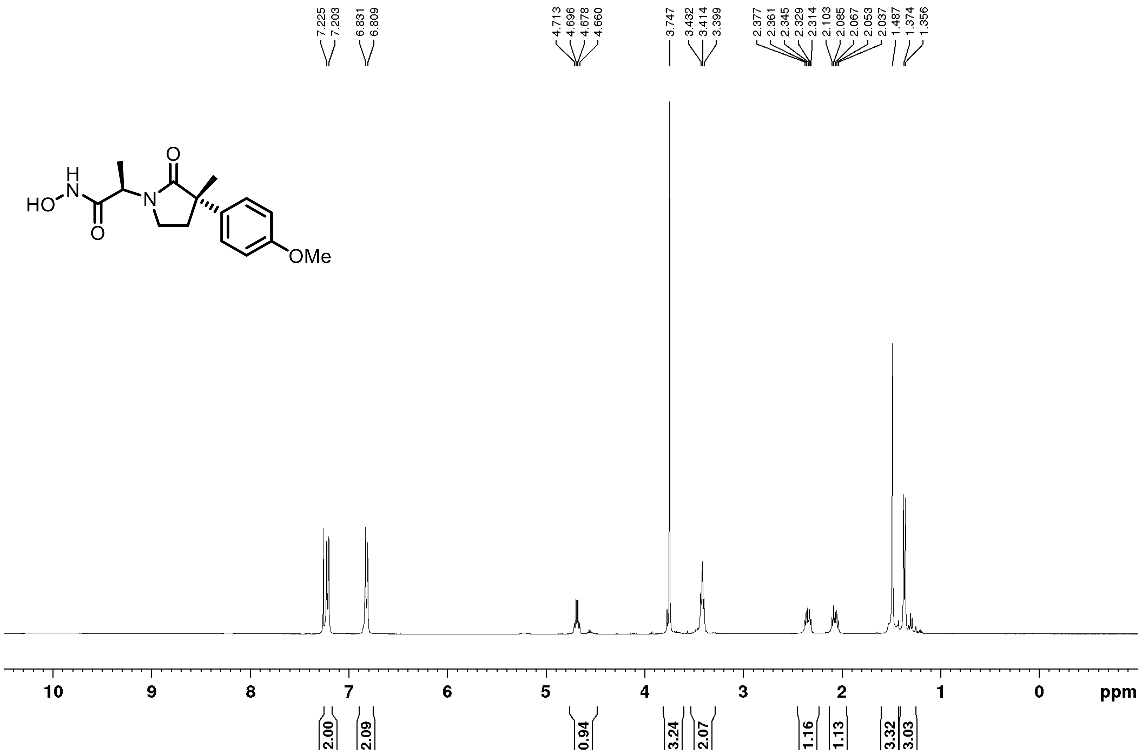

^{13}C (125 MHz, CDCl$_3$)

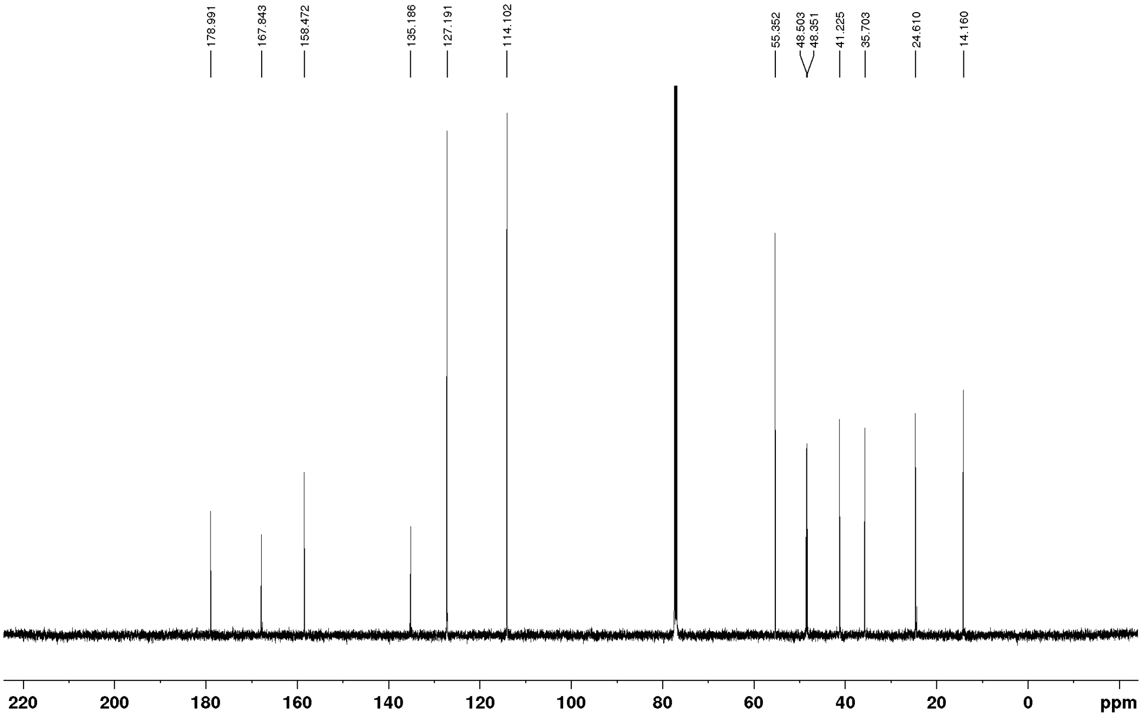

(*R*)-*N*-hydroxy-2-((*S*)-3-(4-chlorophenyl)-3-methyl-2-oxopyrrolidin-1-yl)propenamide (6)

^{1}H (400 MHz, CDCl$_3$)

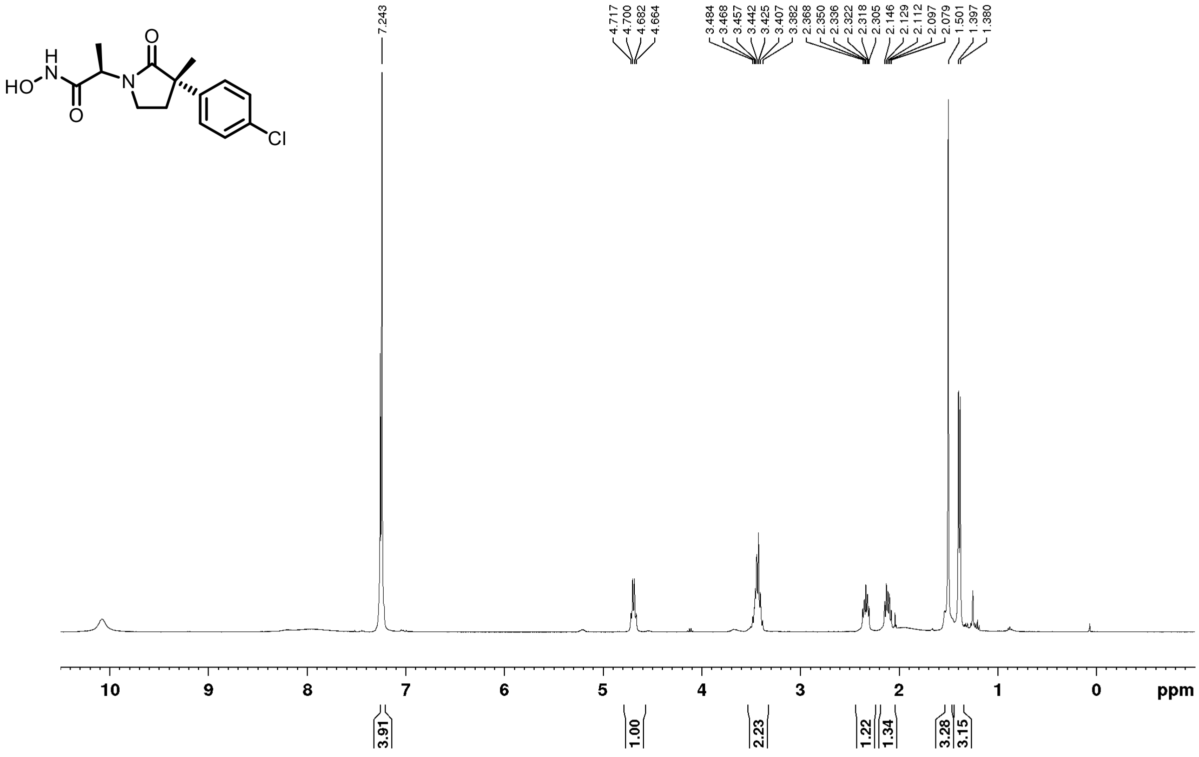

^{13}C (125 MHz, CDCl$_3$)

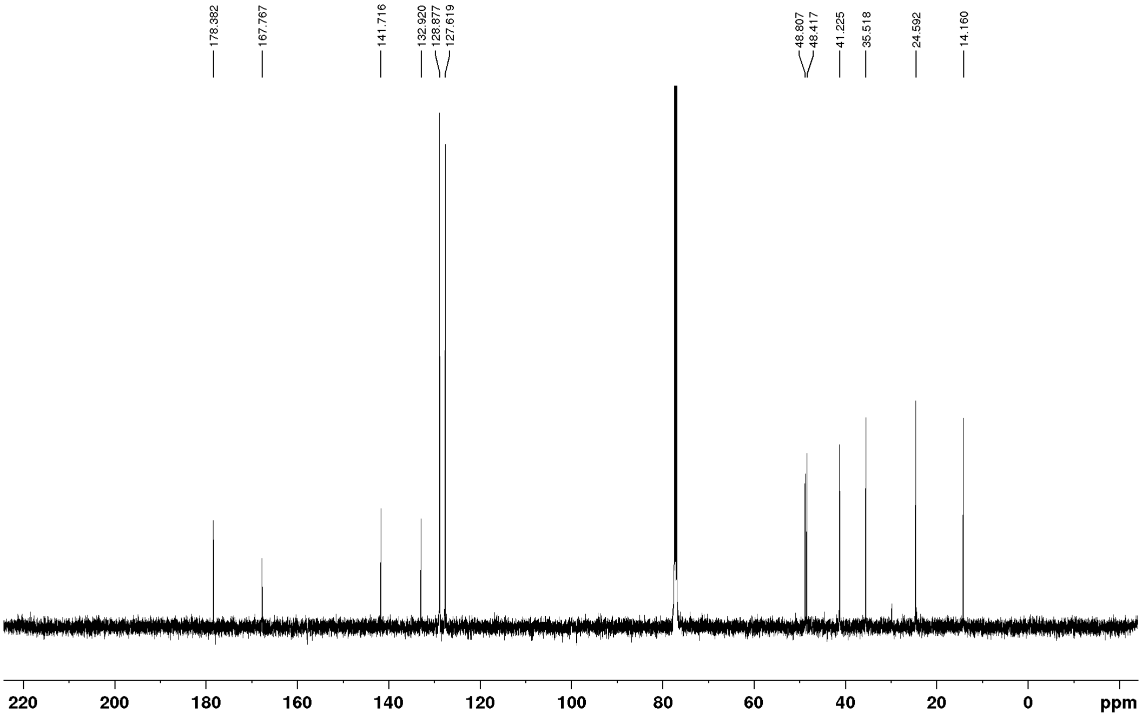

(*R*)-*N*-hydroxy-2-((*S*)-3-(*p*-tolyl)-3-methyl-2-oxopyrrolidin-1-yl)propenamide (7)

^{1}H (400 MHz, $CDCl_3$)

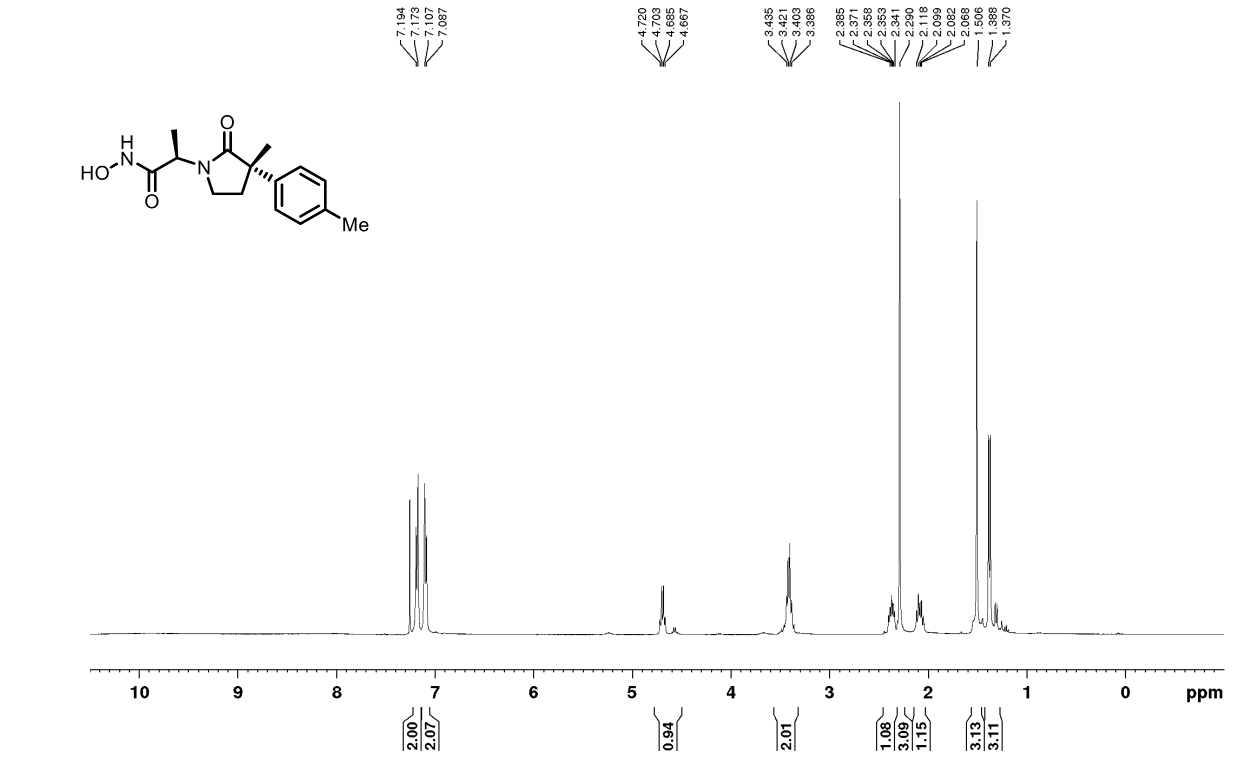

^{13}C (125 MHz, $CDCl_3$)

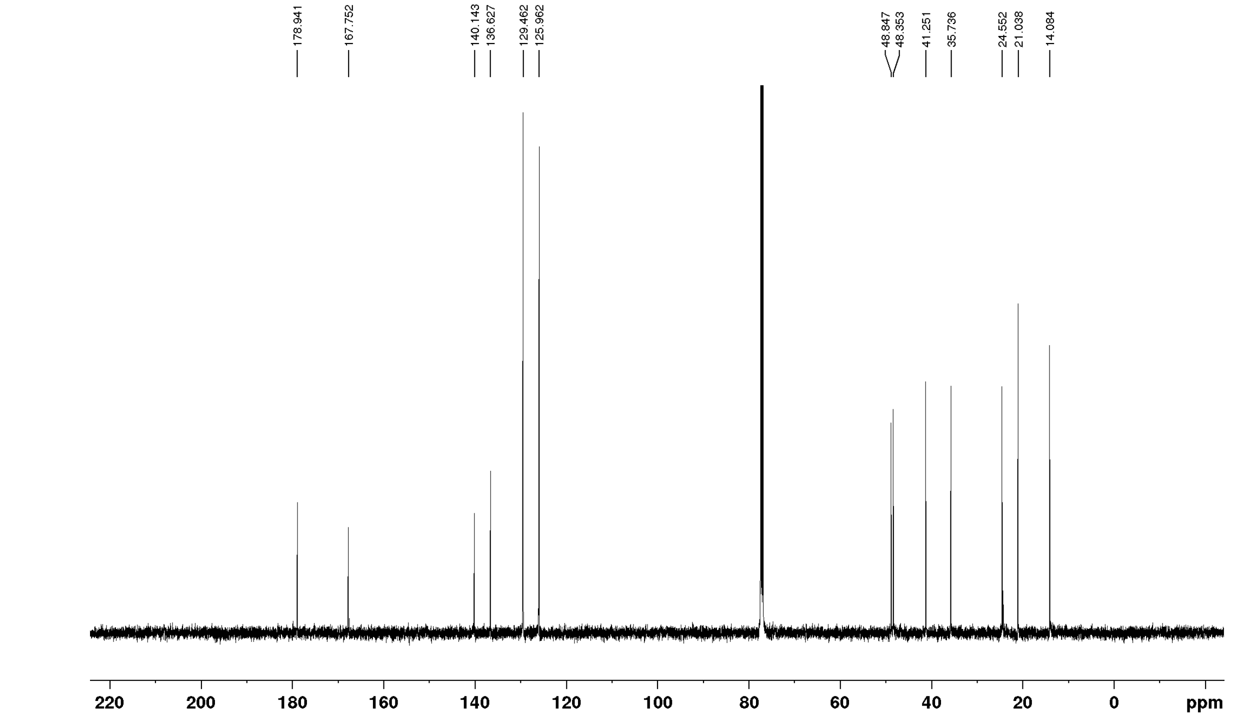

3. Purity analysis of tested compounds by HPLC

(*R*)-*N*-hydroxy-2-((*S*)-3-(4-methoxyphenyl)-3-methyl-2-oxopyrrolidin-1-yl)propenamide (5)

GL science Inertsil ODS-3, 5 μm, 4.6 × 75 mm (0.1% TFA in H_2O / 0.1% TFA in MeCN = 78:22, flow rate = 1.0 mL/min, 254 nm)

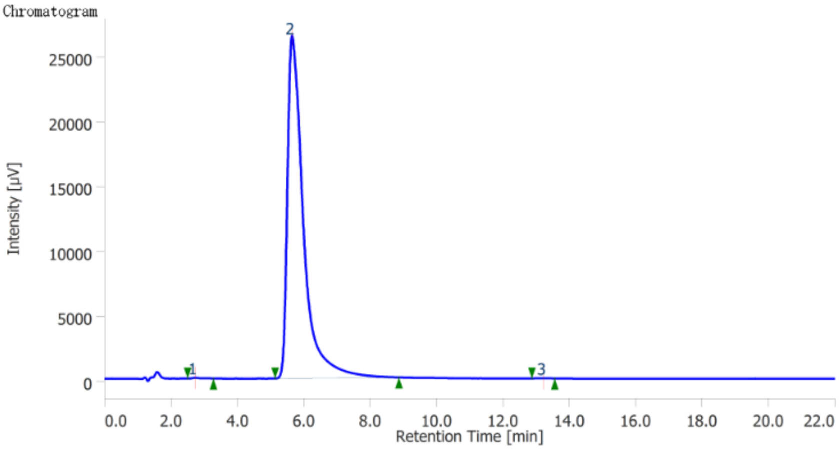

Peak Information

#	tR [min]	Area [μV·sec]	Height [μV]	Area%	Height%
1	2.717	866	51	0.098	0.193
2	5.650	886287	26412	99.864	99.746
3	13.225	341	16	0.038	0.060

(*R*)-*N*-hydroxy-2-((*S*)-3-(4-chlorophenyl)-3-methyl-2-oxopyrrolidin-1-yl)propenamide (6)

GL science Inertsil ODS-3, 5 μm, 4.6 × 75 mm (0.1% TFA in H_2O / 0.1% TFA in MeCN = 73:27, flow rate = 1.0 mL/min, 254 nm)

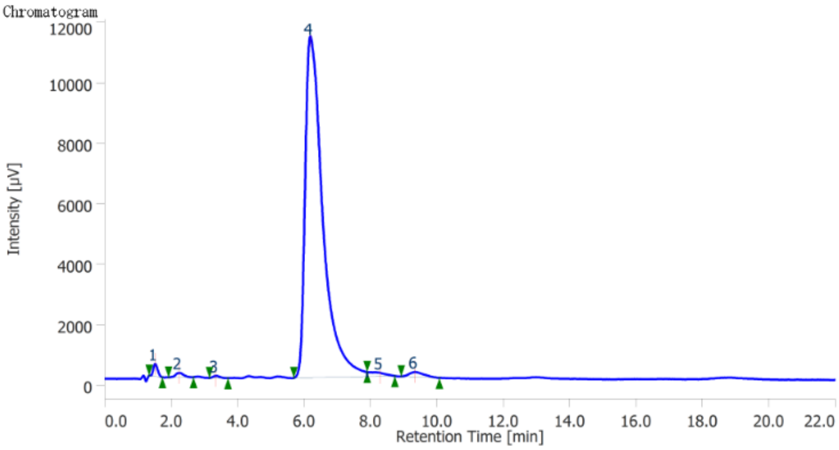

Peak Information

#	tR [min]	Area [μV·sec]	Height [μV]	Area%	Height%
1	1.517	3780	412	0.871	3.382
2	2.242	2518	147	0.580	1.208
3	3.342	996	70	0.230	0.572
4	6.192	417132	11285	96.121	92.601
5	8.300	4521	111	1.042	0.912
6	9.342	5021	161	1.157	1.325

(*R*)-*N*-hydroxy-2-((*S*)-3-(*p*-tolyl)-3-methyl-2-oxopyrrolidin-1-yl)propenamide (6)

(*R*)-*N*-hydroxy-2-((*S*)-3-(4-chlorophenyl)-3-methyl-2-oxopyrrolidin-1-yl)propenamide (7)

GL science Inertsil ODS-3, 5 µm, 4.6 × 75 mm (0.1% TFA in H_2O / 0.1% TFA in MeCN = 73:27, flow rate = 1.0 mL/min, 254 nm)

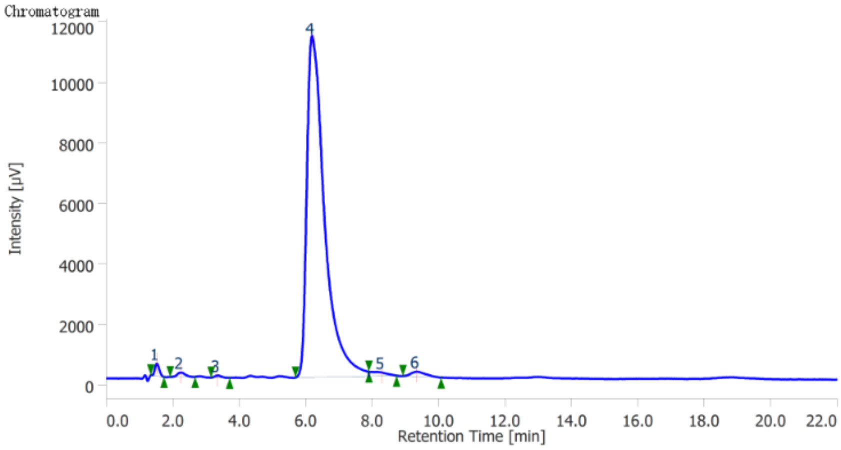

Peak Information

#	tR [min]	Area [µV·sec]	Height [µV]	Area%	Height%
1	1.517	3780	412	0.871	3.382
2	2.242	2518	147	0.580	1.208
3	3.342	996	70	0.230	0.572
4	6.192	417132	11285	96.121	92.601
5	8.300	4521	111	1.042	0.912
6	9.342	5021	161	1.157	1.325

4. MMP1 inhibitory activity of compounds at tested concentration

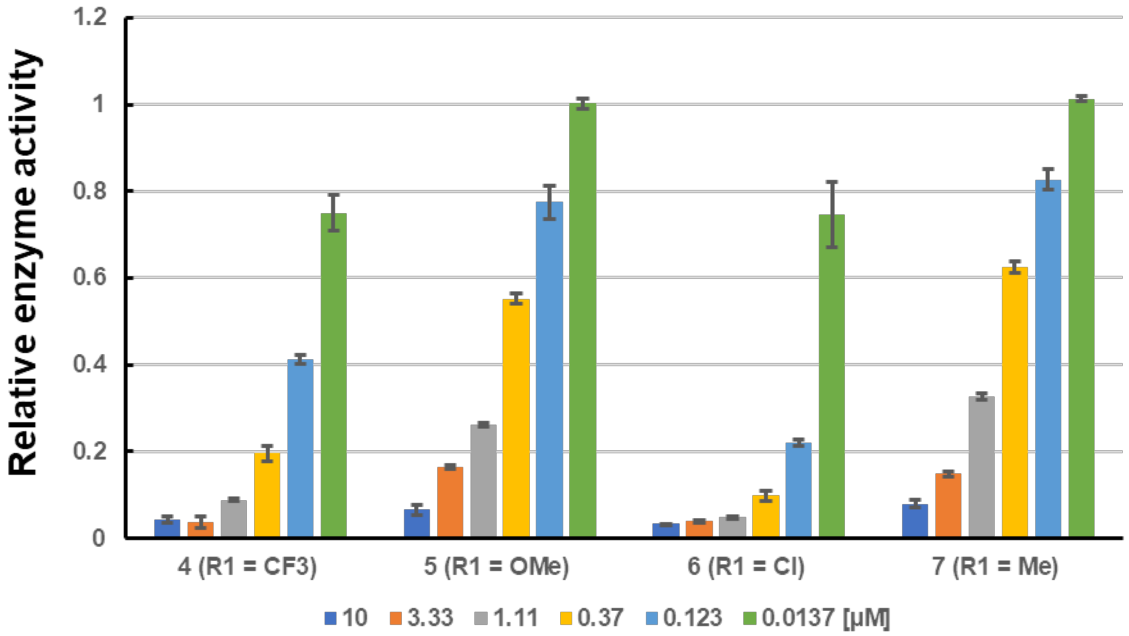

Relative activity of MMP1 of compounds at indicated concentrations are shown as means ± standard error of the mean (SEM) from three independent experiments.
